# Evaluation of Different Bioreactors During In Vitro Shoot Proliferation and Acclimatization of Agave (*Agave marmorata* Roezl)

**DOI:** 10.3390/plants15071054

**Published:** 2026-03-30

**Authors:** María del Rosario Moreno-Hernández, Eucario Mancilla-Álvarez, José Abel López-Buenfil, Jericó Jabín Bello-Bello

**Affiliations:** 1 Colegio de Postgraduados Campus Córdoba, Amatlán de los Reyes 94953, Veracruz, Mexico; morenohdez01@gmail.com (M.d.R.M.-H.); euca_man90@hotmail.com (E.M.-Á.); 2 Colegio de Postgraduados Campus Montecillo, Texcoco 56264, Estado de México, Mexico; 3 Secretaría de Ciencia, Humanidades, Tecnología e Innovación, Colegio de Postgraduados Campus Córdoba, Amatlán de los Reyes 94953, Veracruz, Mexico

**Keywords:** temporary immersion systems, in vitro multiplication, chlorophyll, carotenoids, stomata, acclimatization

## Abstract

Temporary Immersion Systems (TISs) are an efficient alternative for in vitro plant regeneration. This study aimed to evaluate the effect of different culture methods on the in vitro shoot proliferation and acclimatization of agave (*Agave marmorata* Roezl). The culture methods compared were a recipient for automated temporary immersion (RITA^®^), a temporary immersion bioreactor (TIB), a SETIS™ bioreactor, and a semisolid medium control. After eight weeks of in vitro culture, the hyperhydricity of the explants, development variables, photosynthetic pigment content, stomatal density, and survival percentage during acclimatization were evaluated. The results showed that TISs significantly reduced explant hyperhydricity and increased the multiplication rate, number of shoots and leaves, number of roots per shoot, root length, carotenoid content, stomatal density, and percentage of closed stomata during in vitro shoot proliferation. Furthermore, TISs resulted in a higher number of leaves and roots and improved the survival percentage during acclimatization compared to the semisolid medium. Explants cultured in the SETIS™ bioreactor showed the highest photosynthetic pigment content. In conclusion, the evaluated TISs enhanced the physiological development of the explants, favoring the multiplication rate and survival percentage during the acclimatization of *A. marmorata*.

## 1. Introduction

Agave (*Agave marmorata* Roezl), belonging to the Asparagaceae family, is mainly cultivated for the production of mezcal, a distilled alcoholic beverage with a Mexican designation of origin [1]. Additionally, some of the leaves and inflorescences of this species are used for decoration at religious events [2]. This species reproduces sexually by seeds and asexually by suckers; however, seed availability is limited due to intensive harvesting from plantations before flowering [3]. Furthermore, the seeds have a low germination rate (0.42%) [4]. Asexual reproduction produces a small number of suckers, which restricts vegetative propagation [5]. Given this situation, in vitro plant propagation is a viable alternative to increase the production of plants with high genetic and phytosanitary quality in the short term under aseptic and controlled conditions [6].

Several reports have been published on the micropropagation of the genus *Agave*: *A. guiengola* [7], *A. wocomahi* [8], *A. maximiliana* [9], *A. tequilana* [10], *A. potatorum* [11], and *A. sisalana* [12]. However, these protocols utilize semisolid medium, which increases production costs due to (1) expensive gelling agents, (2) high labor costs, and (3) the lack of semi-automation during micropropagation [13]. The in vitro regeneration of *Agave* spp. in temporary immersion systems (TISs) has been investigated in *A. angustifolia* [14], *A. tequilana* [10], *A. potatorum* [15], *A. angustifolia* [16], *A. marmorata* and *A. potatorum* [17], *A. potatorum* [18], and *A. marmorata* [5]. TISs are containers designed for semi-automated, commercial-scale micropropagation under controlled conditions. By enhancing the availability of nutrients, growth regulators, vitamins, and other organic compounds, these systems boost in vitro multiplication rates and reduce production costs while improving efficiency and biological performance [13]. Furthermore, TISs that operate via airflow can stimulate photosynthesis, respiration, and stomatal function [6]. Additionally, different TISs can affect explant hygroscopicity, increase biomass proliferation, enhance stomatal function, promote photosynthetic pigment synthesis, and improve survival during acclimatization.

Several commercially available air-flow TISs have been evaluated for plant micropropagation, including the Recipient for Automated Temporary Immersion (RITA^®^) [19], the Temporary Immersion Bioreactor (TIB) [20], the Ebb-and-Flow Bioreactor [21], the Modular Immersion Bioreactor (BioMINT II™) [22], and the SETIS™ Bioreactor [23]. Comparing different bioreactors during in vitro propagation is important due to the cost and availability of each system, as well as the effect of bioreactors on the physiological and biochemical processes of in vitro explants. This study aimed to evaluate the effect of different culture methods on the in vitro shoot proliferation and acclimatization of *A. marmorata*.

## 2. Results

### 2.1. Effects of Different Culture Systems on Shoot Regeneration

**Figure 1:**
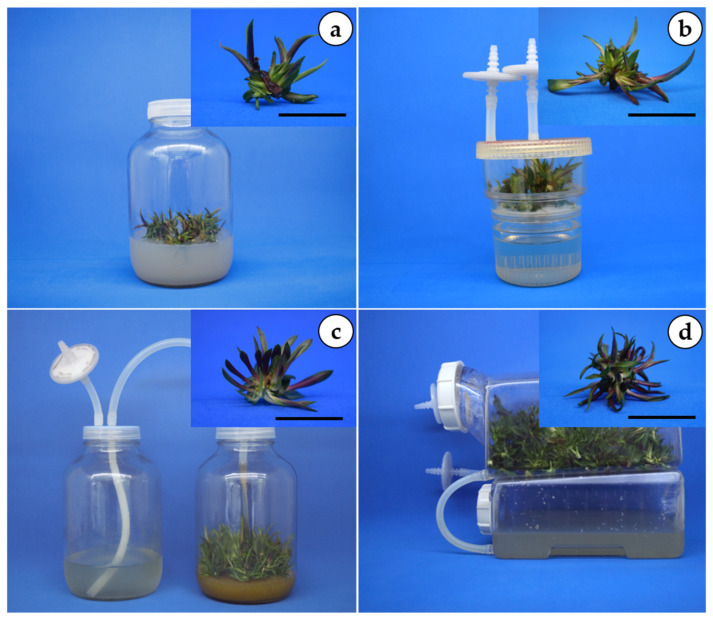
Effects of different culture conditions on in vitro multiplication of *Agave marmorata* explants after eight weeks. (**a**) Semisolid medium, (**b**) RITA, (**c**) TIB, and (**d**) SETIS. RITA: recipient for automated temporary immersion; TIB: temporary immersion bioreactor; SETIS: SETIS™ bioreactor. Black bars = 1 cm.

The results showed different effects on hyperhydricity, shoots per explant, leaves per shoot, and shoot length among the culture methods evaluated during the in vitro propagation phase of *A. marmorata* (Table 1). The semisolid system exhibited the highest hyperhydricity (45.77%), whereas the TIB and SETIS™ systems recorded the lowest (11.56% and 11.75%, respectively). The TISs also yielded the highest number of shoots (averages ranging from 18.80–22.10) and leaves (2.92–3.15) per explant, roughly doubling the output of the semisolid system (9 shoots and 2.47 leaves per explant). While the longest shoots were found in the TIB and SETIS™ systems (2.69 and 2.68 cm, respectively), the RITA^®^ produced the shortest (2.33 cm). Regarding root development, the TISs again outperformed other methods, averaging 1.20–1.80 roots per shoot (1.12–1.40 cm long), while the semisolid system failed to produce any roots. Figure 1 illustrates the effect of the different culture methods on in vitro regeneration of agave.

### 2.2. Effect of Culture Methods on Chlorophyll, β-Carotene, and Lycopene Content

**Figure 2:**
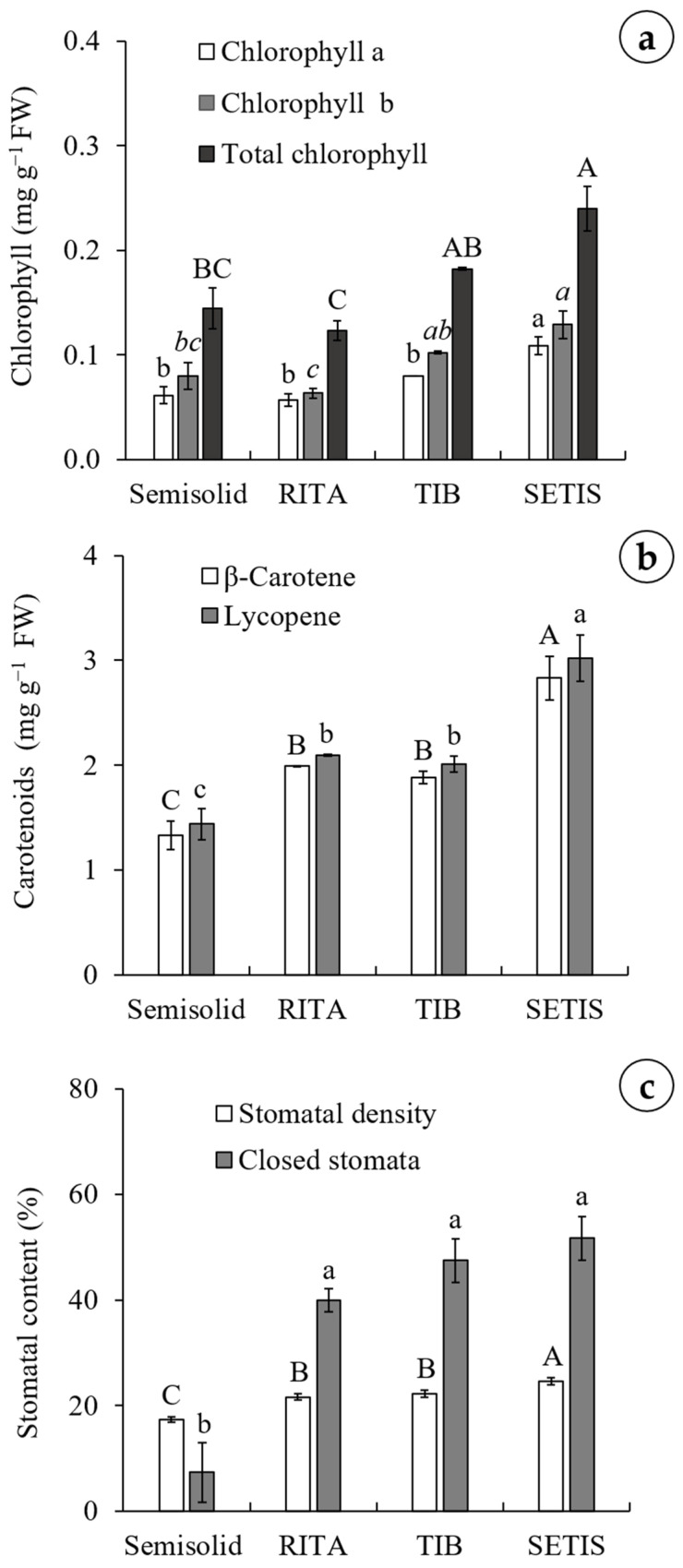
Effects of different culture methods on in vitro regeneration of agave (*Agave marmorata* Roezl) after eight weeks. (**a**) Chlorophyll content, (**b**) carotenoid content, and (**c**) stomatal content. The values represent the means ± standard error. Different letters represent a significant difference according to Tukey’s test (*p* < 0.05). FW: fresh weight; RITA: recipient for automated temporary immersion; TIB: temporary immersion bioreactor; SETIS: SETIS™ bioreactor.

Chlorophyll (a, b and total) and carotenoids (*β*-carotene and lycopene) levels varied significantly across the evaluated culture methods (Figure 2a,b). The SETIS^™^ bioreactor consistently yielded the highest concentrations across all parameters. Specifically, it produced the highest chlorophyll a (0.10 mg g^−1^ FW), while the lowest levels were found in the RITA^®^ (0.05 mg g^−1^ FW), semisolid medium (0.06 mg g^−1^ FW), and TIB (0.08 mg g^−1^ FW). Similarly, chlorophyll b and total chlorophyll peaked in the SETIS^™^ bioreactor at 0.12 and 0.24 mg g^−1^ FW, respectively, nearly doubling the lowest values recorded in the RITA^®^ system (0.06 mg g^−1^ FW and 0.12 mg g^−1^ FW for chlorophyll b and total chlorophyll, respectively).

Carotenoid content followed a similar trend. The SETIS™ bioreactor reached the highest levels of β-carotene (2.83 mg g^−1^ FW) and lycopene (3.02 mg g^−1^ FW). Conversely, the lowest concentrations were observed in the semisolid system, which yielded 1.33 mg g^−1^ FW of β-carotene and 1.44 mg g^−1^ FW of lycopene.

### 2.3. Stomatal Density and Percentage of Closed Stomata

Significant differences in stomatal density (SD) and percentage of closed stomata (CS) were observed among the culture methods (Figure 2c). The maximum value percentage SD was observed in the SETIS™ bioreactor with 24.66%, while the minimum value (17.33%) was found in the semisolid system. Regarding percentage CS, the maximum values were observed in the TISs, ranging from 39.99–51.72%, while the minimum value was found in the semisolid culture system, with 7.40%.

### 2.4. Acclimatization of Agave Plantlets

**Figure 3:**
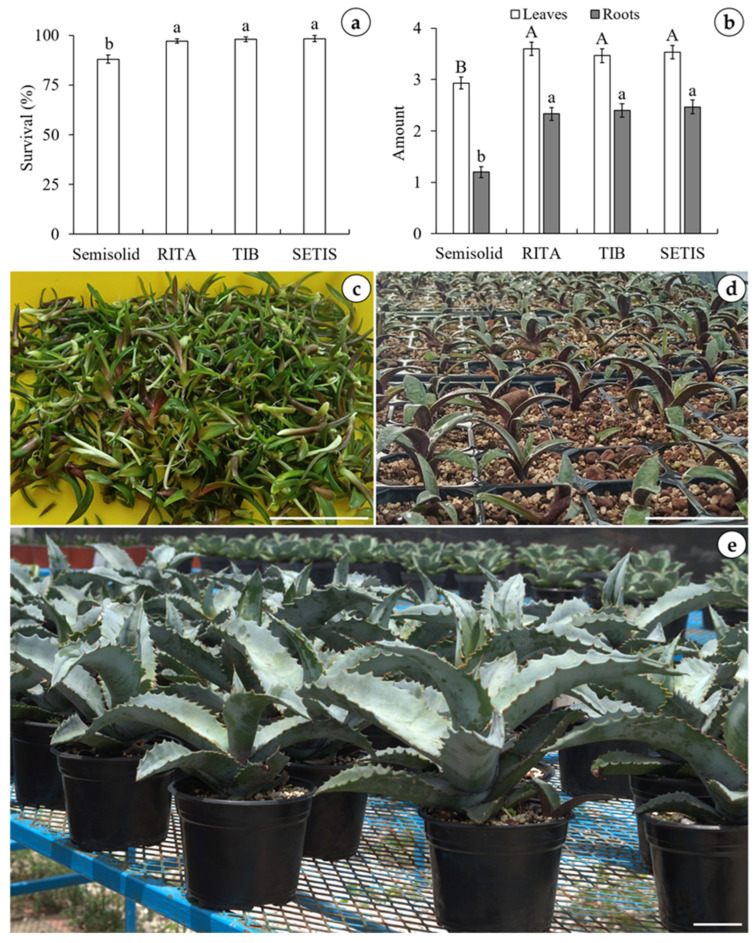
Acclimatization of agave (*Agave marmorata*) plantlets obtained from the different culture methods. (**a**) Survival percentage of plantlets after four weeks in the greenhouse, (**b**) number of leaves and roots per plantlet after eight weeks in the greenhouse, (**c**) plantlets after eight weeks of in vitro culture using different culture methods, (**d**) plantlets after four weeks in the greenhouse, and (**e**) plants transplanted into pots and placed outdoors after 96 weeks. RITA: recipient for automated temporary immersion; TIB: temporary immersion bioreactor; SETIS: SETIS™ bioreactor. Different letters represent a significant difference according to Tukey’s test (*p* < 0.05).

Under ex vitro conditions, significant differences in survival percentages were observed among the different culture methods evaluated. The highest survival rates were achieved using the TISs (97.14–98.33%), while the minimum was recorded in the semisolid medium at 88.00% (Figure 3a). Similarly, the TISs yielded the highest number of leaves, averaging 3.46–3.60 per plantlet, compared to only 2.93 in the semisolid system. Rooting followed a similar trend; plantlets in the TISs produced the highest average of 2.33–2.46 roots, whereas those in the semisolid system had the lowest average of 1.20 (Figure 3b). The acclimatization of *Agave marmorata* is shown in Figure 3c–e.

## 3. Discussion

### 3.1. In Vitro Propagation Using Different Culture Methods

Overall, the evaluation of the different culture methods showed that the TISs promoted better in vitro development of *A. marmorata* compared to the semisolid medium. The TISs had an effect on hyperhydricity, shoots per explant, leaves per shoot, and shoot length. The higher hyperhydricity observed in the semisolid medium could be due to the high relative humidity caused by the airtight seal of the culture vessels [24]. Conversely, the decreased hyperhydricity in the TIS-grown shoots was probably due to the frequency and duration of immersion, indicating adequate ventilation and absorption of water and culture medium components. The immersion time and frequency (2 min every 8 h) have proven favorable in previous studies of *A. marmorata* [5,25]. Among the TISs evaluated, the RITA^®^ system showed increased shoot hyperhydricity, likely due to low light irradiance and high relative humidity. This could be because RITA^®^, made from polyurethane, has a translucent, non-transparent color. Furthermore, in this system, the culture medium and explants are in the same container, resulting in high relative humidity. Hyperhydricity is a physiological disorder that can occur due to the type of growth regulators used, low light irradiance, excess nutrients, high relative humidity within the culture containers, and high water availability [21,26].

The increased in vitro multiplication rate of agave in TISs can be attributed to two factors: (1) greater availability of nutrients, vitamins, sucrose, and growth regulators in the culture medium, and (2) the loss of apical dominance caused by explant movement within the bioreactor. This physiological effect results from low or null auxin synthesis or the reduced transport of these hormones [13]. These advantages are important for commercial micropropagation of plants using a TIS. The application of TISs significantly enhanced shoot proliferation in *Agave* spp. under in vitro conditions, as confirmed in the TIB [7], Ebb-and-Flow bioreactor [21], RITA^®^ [15], and SETIS™ bioreactor systems [18]. Chávez-Ortiz et al. [7] reported that *A. guiengola* produced 43.0 shoots per explant during in vitro multiplication in the TIB, compared to 11.7 shoots per explant in semisolid medium. Similarly, Correa-Hernández et al. [21] found that *A. potatorum* yielded 27.71 shoots per explant during in vitro propagation in an Ebb-and-Flow bioreactor versus 6.27 in semisolid medium. Castillo-Martínez et al. [15] developed an in vitro propagation protocol for *A. potatorum* using the RITA^®^ system, achieving 14.4 shoots per explant compared to 6.4 in semisolid medium. Finally, Mancilla-Álvarez et al. [18] observed significant differences in *A. potatorum* during in vitro propagation using the SETIS™ bioreactor, obtaining 17.33 shoots per explant compared to 6.75 in semisolid medium. Regarding leaf count, TIS treatments yielded the maximum number of leaves per shoot, likely due to increased multiplication rates and the ample space between explants in the bioreactor. Minchala-Buestán et al. [6] demonstrated that shoots obtained in TISs are more vigorous than those grown in a semisolid medium. Shoot length per explant was greatest in the SETIS™ bioreactor and TIB, which could be due to the height of the space within the container. In the RITA^®^, the headspace is limited due to a smaller surface area, which could explain the formation of smaller shoots.

### 3.2. Photosynthetic Pigment Content (Chlorophyll and Carotenoids)

Shoots cultured in the different TISs exhibited higher contents of chlorophylls (Chl a, b, and total) and carotenoids (*β*-carotene and lycopene) compared to those in the semisolid culture medium. The high chlorophyll and carotenoid content in the SETIS™ bioreactor could be due to its horizontal design and completely transparent polycarbonate construction. These features enhance the amount of light irradiance reaching the shoots, which is an important factor that controls the development of explants by influencing the content of photosynthetic pigments, particularly when compared to vertical systems like RITA and TIB [27]. Chlorophyll serves as an indirect indicator of the photosynthetic rate of explants under in vitro conditions [21]. Chl a is the primary pigment responsible for converting light into chemical energy, while Chl b is an accessory pigment that captures light, transfers energy to Chl a, and supports photoprotection. On the other hand, carotenoids are molecules that act as antioxidants and absorb excess light from chlorophylls for the photoprotection of photosystems I and II [28,29]. *β*-carotene is an orange carotenoid that acts as an important protectant for plants against photooxidative damage by quenching toxic oxygen species caused by ultraviolet rays [30,31]. Lycopene, on the other hand, is a pigment that participates in light absorption, photoprotection, and pigmentation [32].

The SETIS™ bioreactor reduces competition among explants, improving light interception. Increased light availability, in turn, stimulates carotenoid biosynthesis through activation of the isoprenoid pathway and plastid development. Bello-Bello et al. [27] reported an increase in chlorophyll content in banana (*Musa* AAA cv. Grand Naine) after temporary immersion compared to semisolid medium. Hwang et al. [33] in chrysanthemum (*Chrysanthemum morifolium)* and strawberry *(Fragaria × ananassa* Duch.) reported a high content of photosynthetic pigments when using the SETIS™ bioreactor compared to semisolid medium. Mancilla-Álvarez et al. [18] observed an increase in chlorophyll content in agave tobalá (*A. potatorum*) when using the SETIS™ and MATIS^®^ systems, compared to semisolid medium. On the other hand, an increase in *β*-carotene content has been reported in TISs in banana (*Rasthali* AAB—Silk) [34], and gerbera (*Gerbera jemesonii*) [35]. Uma et al. [34] observed an increase in *β*-carotene in banana (*Rasthali* AAB—Silk) when using the TIB. Lastly, Lim et al. [35] reported an increase in carotenoid content in *G. jemesonii* when using the SETIS™ bioreactor.

### 3.3. Stomatal Content

In this study, stomatal density (SD) and percentage closed stomata (CS) were affected by the different culture methods evaluated. Stomata regulate gas exchange, transpiration, and respiration; they are essential for regulating water potential in tissues, thus contributing to the maintenance of tissue homeostasis [36]. The SETIS™ bioreactor system showed an increase in SD compared to the other culture methods, likely due to greater transpiration, lower relative humidity in the culture vessel, and better light distribution within this bioreactor. These factors may promote stomatal formation during leaf development.

SD, which quantifies the number of stomata per unit of leaf area, is one of several plant traits affecting leaf gas exchange. It is influenced by multiple environmental and physiological factors, including light irradiance, relative humidity, tissue nutritional status, and oxygen and carbon dioxide [18,37]. The high percentage CS in the TISs suggests adequate stomatal function when using bioreactors. This is likely because the availability of O_2_, CO_2_ and other gases supports the stomatal function of the explant leaves [38]. In addition, nutrient availability could favor stomatal function. Nitrogen (N) and magnesium (Mg) are structural components of chloroplasts within the stomata, while potassium (K) regulates stomatal opening and closing via the potassium pump [39].

Phosphorus (P) availability has also been linked to changes in stomatal conductance [40]. Additionally, plants close their stomata to regulate the consumption of available O_2_ [41], indicating adequate stomatal function. Vendrame et al. [38] note that stomata dynamically regulate plant water status and gas exchange during photosynthesis, a process that may enhance chlorophyll synthesis. Similarly, Solís-Zanotelli et al. [42] point out that stomatal function and the gas exchange mechanism in plants cultured in TISs improve photomixotrophic capacity and could prepare explants for future ex vitro conditions during acclimatization. Conversely, the low SD and percentage CS in the semisolid culture was probably due to the increase in the percentage of shoot hyperhydricity caused by high relative humidity in the culture vessels. Luomala et al. [43] and de Oliveira et al. [44] note that cultures in semisolid media have no or limited gas exchange, decreasing CO_2_ and O_2_ levels in the shoots, which can inhibit stomatal development and function.

### 3.4. Acclimatization of Agave Plantlets

Acclimatization is the process by which in vitro explants adapt to ex vitro conditions. In this study, TISs exhibited high survival rates (97.14–98.33%) during the acclimatization phase, demonstrating the efficiency of *A. marmorata* micropropagation protocols when using bioreactors. Martínez-Martínez et al. [25] have also reported high survival rates during the acclimatization of *A. marmorata* plantlets using TISs. These favorable outcomes in plants obtained via TISs could be related to enhanced gas exchange, improved stomatal function, and the synthesis of photosynthetic pigments prior to acclimatization.

## 4. Materials and Methods

### 4.1. In Vitro Establishment and Shoot Regeneration

*A. marmorata* explants were previously established according to the protocol of Mancilla-Álvarez et al. [5] using MS culture medium [45] containing 3 mg L^−1^ 6-benzylaminopurine, 2 mg L^−1^ indolacetic acid, and 0.8% agar (PhytoTech Labs, Lenexa, KS, USA) as a gelling agent. The culture medium pH was adjusted to 5.7 ± 0.2. Culture vessels and bioreactors were sterilized in an autoclave (CVQ-B75L, Ecoshel, Pharr, TX, USA) at 1.5 kg cm^−2^ and 121 °C for 25 min. Explants were maintained in an incubation room at 25 ± 2 °C under warm white LED lamps (50 ± 5 μmol m^−2^ s^−1^) with a 16:8 h (light–dark) photoperiod.

### 4.2. Evaluation of Different Culture Methods

Shoots generated were used as explants to evaluate different culture methods: conventional propagation in semisolid and liquid medium in RITA^®^, TIB and the SETIS™ bioreactor. The number of explants used in each cultivation system is according to its headspace. All culture systems were maintained under equal conditions per container, using 200 mL of headspace volume and 50 mL of medium per explant during the in vitro multiplication phase. The immersion frequency was 2 min every 8 h, according to an optimized protocol for *Agave marmorata* described by Mancilla-Álvarez et al. [5]. For RITA^®^, five explants were placed per container. For the semisolid and TIB methods, 2000 mL glass containers were used, holding 10 explants each. For the TIB, a polyurethane sponge was included to ensure the correct immersion frequency and time. In the SETIS™ bioreactor, 29 explants were cultured per container. The experimental design included four containers per treatment; specifically, four containers were used for semisolid medium and four bioreactors for each TIS. After eight weeks of culture, the following variables were evaluated for each culture system: hyperhydricity percentage, number of shoots and leaves, number of roots per shoots, root length, chlorophyll, carotenoid, and stomatal content. In addition, number of leaves and roots per plantlet were recorded, as well as survival percentage during the acclimatization.

### 4.3. Photosynthetic Pigment Content

Chlorophyll (Chl) content. The content of chlorophylls (Chl a, Chl b and total chlorophyll) was determined using the protocol described by Mahmood et al. [46] with modifications. Total chlorophyll (Chl t) content is expressed as Chl a + b. For each culture system, 500 mg of fresh leaves tissue was homogenized in a mortar with 5 mL of 70% acetone. The samples were stored under dark conditions in amber bottles for 24 h at a temperature of −4 °C. Subsequently, the extract was filtered using No. 41 filter paper and the volume adjusted to 12.5 mL with 80% acetone. The absorbance of the samples was determined using a spectrophotometer (Lambda 35, PerkinElmer^®^, Waltham, MA, USA) at 663 and 645 nm for chlorophyll a and b, respectively.

$$\left(Chl\;a\right)\;\left(mg\cdot g^{-1}\right)=\frac{\left(12.72\times OD663-2.59\times OD645\right)\times V}{1000\times W}$$

$$\left(Chl\;b\right)\;\left(mg\cdot g^{-1}\right)=\frac{\left(22.88\times OD645-2.59\times OD663\right)\times V}{1000\times W}$$

Total chlorophyll content (mg g^−1^) = *Chl a* + *Chl b*

where OD_663_ and OD_645_: absorbance, V: volume graduation in mL^−1^, W: sample weight in g, 1000: conversion factor.

*β*-carotene content. *β*-carotene content was determined according to the methodology described by Biehler et al. [47]. For each culture system, 2 mL of extract in 70% acetone was used. Absorbance was determined using a spectrophotometer (Lambda 35, PerkinElmer^®^) at 450 nm.

Lycopene content. Lycopene content was assessed following the methodology of Pal et al. [48]. For each culture system, 0.25 g of fresh leaf tissue was weighed and macerated by adding 5 mL of 80% acetone. Absorbance was recorded using a spectrophotometer (Lambda 35, PerkinElmer^®^) at 470 nm.

### 4.4. Stomatal Density and Percentage of Closed Stomata

Stomatal density (SD) and the percentage of closed stomata (CS) were estimated as described by Xu and Zhou [49]. Samples were taken from the abaxial leaf surface. Epidermal cells per mm^2^ were calculated from five random leaf fields and observed using a compound microscope (Carl Zeiss MicroImaging, GmbH 37081, Göttingen, DEU, Germany). For each culture system, SD was calculated using the following formula:

$$\;Stomatal\;density\;=\frac{Number\;of\;stomata}{Number\;of\;epidermal\;cells+number\;of\;stomata}\times 100$$

The percentage of closed stomata was calculated with the formula:

$$Percentage\;of\;closed\;stomata=\frac{Number\;of\;closed\;stomata}{Total\;number\;of\;stomata}\times 100$$

### 4.5. Hyperhydricity Rate

Shoots exhibiting translucent and fragile leaves were classified as hyperhydric explants. After eight weeks of culture, the hyperhydricity percentage was calculated as follows:

$$Hyyperhydricity\;percentage=\frac{Hyperhydric\;shoots}{Normal\;shoots}\times 100$$

### 4.6. Acclimatization of Agave Plantlets

To determine the survival percentage, 50 shoots, each 2–3 cm long, were taken from the different culture system tested. To prevent contamination of the ex vitro plantlets, the substrate was sterilized in an autoclave at 120 °C and 110 kPa for 25 min. The shoots were transplanted into 50-cell cavity trays containing a substrate consisting of tezontle, soil, and agrolite (2:1:1 *v*/*v*/*v*). The shoots were covered with a translucent polyvinyl chloride dome to control humidity. Plantlets were maintained in a greenhouse (natural light irradiance of 200 µmol m^−2^ s^−1^, 35 ± 5 °C, and 70 ± 5% relative humidity). Throughout the experiment, the plantlets were not fertilized but were irrigated with distilled water three times a week. After 30 d, the survival rate of each culture system was analyzed.

### 4.7. Statistical Analysis

All experiments were conducted in a completely randomized design and replicated three times. The experimental design included four containers per treatment; specifically, four containers were used for the semisolid medium, and four bioreactors were used for each TIS. After eight weeks of culture, the hyperhydricity of the explants, development variables, photosynthetic pigment content, stomatal density, and survival percentage were evaluated for each culture method. To evaluate the assumptions of normality and homogeneity, we conducted the Shapiro–Wilk and Bartlett’s tests, respectively. Data were subjected to analysis of variance and Tukey’s test (*p* < 0.05) using the statistical software package for the social sciences (SPSS), v22 for Windows.

## 5. Conclusions

This study demonstrates that temporary immersion systems have a physiological effect on *Agave marmorata* shoot proliferation and acclimatization. The shoot rooting phase was not necessary, which offers advantages in terms of decreased labor, reagent use, and energy costs, as well as reduced culture time. The temporary immersion systems evaluated are a viable alternative for increasing the multiplication rate and survival during the acclimatization phase of commercial micropropagation of this species.

## Figures and Tables

**Table 1:** Effects of different culture methods on in vitro regeneration of agave (*Agave marmorata* Roezl) after eight weeks of culture.

Culture Methods	Hyperhydricity (%)	Shoots/Explant	Leaves/Shoot	Shoot Length (cm)	Roots/Shoot	Root Length (cm)
Semisolid	45.77 ± 3. 52 a	9.00 ± 0.77 b	2.47 ± 0.07 b	2.48 ± 0.08 ab	0.00 ± 0.00 b	0.00 ± 0.00 b
RITA	24.63 ± 2.77 b	18.80 ± 0.94 a	3.15 ± 0.12 a	2.33 ± 0.08 b	1.20 ± 0.24 a	1.40 ± 0.26 a
TIB	11.56 ± 0.78 c	19.50 ± 1.33 a	2.92 ± 0.13 a	2.69 ± 0.08 a	1.46 ± 0.23 a	1.12 ± 0.27 a
SETIS	11.75 ± 0.85 c	22.10 ± 0. 91 a	2.95 ± 0.10 a	2.68 ± 0.09 a	1.80 ± 0.22 a	1.12 ± 0.21 a

Values represent the mean ± standard error. Means with different letters within a column are significantly different using Tukey’s test (*p* ≤ 0.05). RITA: recipient for automated temporary immersion; TIB: temporary immersion bioreactor; SETIS: SETIS™ bioreactor.

## Data Availability

The data can be made available upon prior request.
